# Dietary berberine alleviates high carbohydrate diet-induced intestinal damages and improves lipid metabolism in largemouth bass (*Micropterus salmoides*)

**DOI:** 10.3389/fnut.2022.1010859

**Published:** 2022-09-23

**Authors:** Yulong Gong, Qisheng Lu, Yulong Liu, Longwei Xi, Zhimin Zhang, Haokun Liu, Junyan Jin, Yunxia Yang, Xiaoming Zhu, Shouqi Xie, Dong Han

**Affiliations:** ^1^State Key Laboratory of Freshwater Ecology and Biotechnology, Institute of Hydrobiology, Chinese Academy of Sciences, Wuhan, China; ^2^College of Advanced Agricultural Sciences, University of Chinese Academy of Sciences, Beijing, China; ^3^The Innovative Academy of Seed Design, Chinese Academy of Sciences, Wuhan, China; ^4^Hubei Engineering Research Center for Aquatic Animal Nutrition and Feed, Wuhan, China

**Keywords:** berberine, carbohydrate, lipid metabolism, AMPK/SREBP1 cascade, intestine health, largemouth bass

## Abstract

High carbohydrate diet (HCD) causes metabolism disorder and intestinal damages in aquaculture fish. Berberine has been applied to improve obesity, diabetes and NAFLD. However, whether berberine contributes to the alleviation of HCD-induced intestinal damages in aquaculture fish is still unclear. Here we investigated the effects and mechanism of berberine on HCD-induced intestinal damages in largemouth bass (*Micropterus salmoides*). We found dietary berberine (50 mg/kg) improved the physical indexes (VSI and HSI) without affecting the growth performance and survival rate of largemouth bass. Importantly, the results showed that dietary berberine reduced the HCD-induced tissue damages and repaired the barrier in the intestine of largemouth bass. We observed dietary berberine significantly suppressed HCD-induced intestinal apoptosis rate (from 31.21 to 8.35%) and the activity level of Caspase3/9 (*P* < 0.05) by alleviating the inflammation (*il1*β, *il8*, *tgf*β, and IL-6, *P* < 0.05) and ER stress (*atf6*, *xbp1*, *perk*, *eif2*α, *chopa*, *chopb*, and BIP, *P* < 0.05) in largemouth bass. Further results showed that dietary berberine declined the HCD-induced excessive lipogenesis (oil red O area, TG content, *acaca*, *fasn*, *scd*, *ppar*γ, and *srebp1*, *P* < 0.05) and promoted the lipolysis (*hsl*, *lpl*, *cpt1a*, and *cpt2*, *P* < 0.05) *via* activating adenosine monophosphate-activated protein kinase (AMPK, *P* < 0.05) and inhibiting sterol regulatory element-binding protein 1 (SREBP1, *P* < 0.05) in the intestine of largemouth bass. Besides, we also found that dietary berberine significantly promoted the hepatic lipid catabolism (*hsl*, *lpl*, *cpt1a*, and *cpt2*, *P* < 0.05) and glycolysis (*pk* and *ira*, *P* < 0.05) to reduce the systematic lipid deposition in largemouth bass fed with HCD. Therefore, we elucidated that 50 mg/kg dietary berberine alleviated HCD-induced intestinal damages and improved AMPK/SREBP1-mediated lipid metabolism in largemouth bass, and evaluated the feasibility for berberine as an aquafeed additive to enhance the intestinal function of aquaculture species.

## Introduction

Carbohydrates are widely applied in aquafeeds as they are characterized by favorable price-performance ratio and puffing-friendly function (1–3). However, fish exhibits a very limited ability in utilizing dietary carbohydrates (4). As a common metabolic disorder, uncontrollable blood glucose excursion is usually observed in carnivorous fish with high carbohydrate diet (HCD) challenge (2, 5, 6). The long-term HCD ingestion caused supraphysiological lipogenesis and glycogenesis, which ultimately deteriorated to hepatic steatosis and hepatomegaly in some aquaculture species (7, 8). Importantly, previous studies demonstrated that HCD impaired the intestinal health in largemouth bass (*Micropterus salmoides*) (9), Nile tilapia (*Oreochromis niloticus*) (10), and yellow catfish (*Pelteobagrus fulvidraco*) (11). However, mechanism investigations of these intestinal disorders remain insufficient. Developing strategies to improve HCD-induced intestinal damages have been a topical subject.

Intestinal tract is the most important organ in the digestion and absorption of nutrients, including carbohydrates, fatty acids, amino acids, and minerals (12, 13). Notably, dietary factor is the main cause for intestinal disfunctions (14–16). Excessive carbohydrates ingestion caused lipid accumulation and oxidative stress in the intestinal epithelial cells (11, 17). The over-deposition of intracellular lipids caused endoplasmic reticulum (ER) stress, chronic inflammation and cell death in intestine (9, 11, 18). Besides, HCD had thought to be implicated in inflammatory bowel disease (IBD) in human or rodents (19, 20). Interestingly, berberine (BBR), a natural isoquinoline alkaloid isolated from Chinese herbs (*Berberisaristata*, *Coptis Rhizome*, and *Coptischinensis*) (21), has been investigated to improve the intestinal health by modulating the microbiota profile in fish, mammal, and human (22, 23). BBR also shown multiple positive effects on ameliorating diabetes, obesity and non-alcoholic fatty liver disease (NAFLD) (24). Detailed investigations revealed that BBR played key roles in improving glucose and lipid metabolism, enhancing insulin sensitivity and defending oxidative stress (25–27). Therefore, BBR is a potential candidate for alleviating the HCD-induced metabolism disorders in the intestine and liver of fish. Recently, studies in fish also demonstrated that BBR reduced hepatic lipid accumulation, improved glucose metabolism and enhanced antioxidative capacity in largemouth bass (28), blunt snout bream (*Megalobrama amblycephala*) (29, 30), black sea bream (*Acanthopagrus schlegeli*) (31), and grass carp (*Ctenopharyngodon idella*) (32), respectively. However, whether BBR alleviates HCD-induced intestinal damages and disorders remains to be deciphered.

Largemouth bass is a carnivorous freshwater fish, which has been widely cultured in many countries and has become one of the economically valuable species with rapid production growth (33, 34). Previous study demonstrated that largemouth bass displayed a severe intolerance of dietary carbohydrates (35), including intestinal disorders (9). In the present study, we evaluated the effects of dietary BBR on alleviating intestinal damages in the largemouth bass fed with HCD. We found that dietary BBR (50 mg/kg) reduced the HCD-induced morphology damages and repaired the barrier by suppressing the inflammation and ER stress in the intestine of largemouth bass. We further interpreted that these positive effects were due to improved lipid metabolism in the intestine and liver of largemouth bass. These findings propose that BBR is a feasible aquafeed additive for enhancing the intestinal health of aquaculture species.

## Materials and methods

### Experimental diets

Three isolipidic (10.10% crude lipid) and isonitrogenous (45.91% crude protein) diets were formulated, which contained 12% cassava starch (NCD), 20% cassava starch (HCD) and 20% cassava starch adding 50 mg/kg berberine (HCD + BBR), respectively. All the ingredients of each diet were crushed and sieved through a 100-mesh sieve. The BBR (Berberine hydrochloride, Solarbio, Beijing, China) was thoroughly mixed by progressive enlargement method after accurate weighing. Then, all the ingredients were completely mixed and extruded into 2 mm diameter pellets under the following extrusion conditions: feeding section (90°C/5 s), compression section (150°C/5 s), and metering section (120°C/4 s) using a twin-screwed extruder (Jinan Dingrun Machinery CO., LTD., Jinan, China). The pellets were dried in an oven (60°C) and stored at 4°C for 7 weeks. The formulation and approximate composition of the experimental diets were shown in Table 1.

**TABLE 1:** Formulation and proximate compositions of experimental diets.

Ingredients (% dry matter)	NCD	HCD	HCD + BBR
Fish meal^a^	40	40	40
Cottonseed protein concentrated^b^	12.3	12.3	12.3
Soybean meal^c^	8	8	8
Casein^d^	8	8	8
Cassava starch^e^	12	20	20
Microcrystalline cellulose^f^	8.5	0.5	0.495
Fish oil^g^	6	6	6
Sodium carboxymethylcellulose^h^	2	2	2
Monocalcium phosphate^i^	2	2	2
Vitamin and mineral additives^j^	1	1	1
Choline chloride (50%)^k^	0.2	0.2	0.2
Berberine (BBR)^l^	0	0	0.005
**Proximate compositions (% dry matter)**			
Crude protein	45.62	46.73	45.35
Crude lipid	10.03	10.65	10.12
Ash	10.56	10.47	10.31
Moisture	10.22	9.86	10.18

^a^Fish meal: Superprime, TASA Fish Product Co., Ltd., Lima, Peru. ^b^Cottonseed protein concentrated: Xinjiang Jinlan Plant Protein Co., Ltd, Xinjiang, China. ^c^Soybean meal: Qingdao Bohai Agricultural Development Co., Ltd, Qingdao, China. ^d^Casein: Lanzhou Longruan Casein Co., Ltd., Lanzhou, China. ^e^Cassava starch: Wuhan Yiteng Starch Co., Ltd., Wuhan, China. ^f^Microcrystalline cellulose: Shandong Liujia Pharmaceutical Excipients Co., Ltd., Jining, China. ^g^Fish oil: Coland Feed Co., Ltd., Wuhan, China. ^h^Sodium carboxymethylcellulose: Shanghai Ever Bright Enterprise Development Co., Ltd., Shanghai, China. ^i^Monocalcium phosphate: Sinopharm Chemical Reagent Co., Ltd., Shanghai, China. ^j^Vitamin and mineral additives: Guangdong Nutriera Group, Guangzhou, China. Vitamin additives, mg/kg diet: vitamin A 10; vitamin B1 6; vitamin B2 5; vitamin B6 7.5; vitamin B12 (1%) 4; niacinamide 50; ascorbyl calcium phosphate (35%) 500; calcium pantothenate 20; biotin (2%) 2.5; folic acid 5; vitamin E (50%) 200; vitamin K3 10; vitamin D3 5; inositol 100; corn protein powder 75. Mineral additives, mg/kg diet: CuSO_4_⋅5H_2_O 10; FeSO_4_⋅H_2_O 300; ZnSO_4_⋅H_2_O 200; MnSO_4_⋅H_2_O 100; KIO_3_ (10%) 80; Na_2_SeO_3_ (10% Se) 67; CoCl_2_⋅6H_2_O (10% Co) 5; NaCl 100; zeolite 638. ^k^Choline chloride: Guangdong Nutriera Group, Guangzhou, China. ^l^Berberine: Berberine hydrochloride, Beijing Solarbio Science and Technology Co., Ltd., Beijing, China.

### Fish and feeding trial

The experimental largemouth bass were obtained from a fish farm (Ezhou, Hubei, China). Three weeks prior to the feeding trial, largemouth bass were acclimated in a fiber glass cylinder (1325 L) and fed to satiation twice a day at 9:00 and 17:00 with a commercial feed (Wuhan CP Aquatic Co., Ltd., Wuhan, China). The feeding trial was conducted in an indoor recirculating system. After a 24-h fasting, the fish of healthy appearance and similar size (initial body weight: 7.99 ± 0.04 g) were randomly distributed into nine fiber glass tanks (120 L) at a density of 20 fish per tank. Triplicate tanks were randomly assigned to each treatment. During the trial, fish were fed to apparent satiation twice a day at 9:00 and 17:00 for 7 weeks. The photoperiod was 12-h light (8:00–20:00): 12-h dark. The water temperature was recorded daily and maintained at 28.0 ± 1.5°C. Ammonia nitrogen (Ammonia-N), dissolved oxygen (DO) and pH were monitored weekly. The values showed that the concentration of Ammonia-N was below 0.4 mg/kg, the DO was 6.0–7.2 mg/L, and the pH was 6.8–7.4.

### Sample collection and growth performance determination

At the end of the feeding trial, all experimental fish were anesthetized with MS-222 (50 mg/L; Sigma Aldrich Co. LLC., St. Louis, MO, USA) and weighed after a 6-h fasting. Three fish in each tank were randomly selected to measure the body length and weight, then dissected to remove and weigh the visceral mass and the liver, respectively. Other two fish in each tank were randomly selected for sampling the blood, liver, and intestine tissues. The blood samples were collected from the caudal vein by heparinized syringe. Then the blood was centrifuged at 3000 g for 10 min to obtain the plasma and stored at −80°C for further analysis. Immediately, the liver and intestine tissues were removed on ice. A small part of each liver and intestine tissues was fixed by 4% paraformaldehyde or 2.5% glutaraldehyde for histological and ultrastructural observation, while the rest was stored at −80°C for further analysis.

The survival rate, specific growth rate (SGR), condition factor (CF), viscerosomatic index (VSI), and hepatosomatic index (HSI) were calculated as follows:


Survival rate (%) = final number of fish/initial number of fish × 100;

SGR (%/d) = [Ln (final body weight) - Ln (initial body weight)]/days × 100;

CF(g/cm)3=wholebodyweight/(bodylength);3

VSI(%)=visceralweight/wholebodyweight×100;

HSI(%)=liverweight/wholebodyweight×100.

### Total RNA extraction, reverse transcription, and qPCR

The total RNA from intestine and liver tissue was extracted with TRIzol Reagent (Ambion Life Technologies, Carlsbad, CA, USA) according to the product manual, and the quality and concentration of the total RNA were tested according to the method of our previous study (34). The total RNA was then reverse-transcribed with an M-MLV First Strand Synthesis Kit (Invitrogen, Shanghai, China). The obtained cDNA was stored at −20°C for later use. The qPCR was performed on LightCycle 480 II system (Roche, Basel, Switzerland). Each sample was run in duplicate, and the relative expressions were calculated according to Vandesompele et al. (36). The primers of qPCR were designed using the National Center for Biotechnology Information (NCBI) primer BLAST service or from the previous studies (37–42) and shown in Table 2.

**TABLE 2:** Primers used for gene expressions assay by qPCR.

Target genes	Forward (5′-3′)	Reverse (5′-3′)	Accession number/source
*claudin1*	GATCAGAGCCACTACCCCAA	TTCCAAAGCCCTTCATACAGC	XM_038718401.1
*occludin*	CAGCCCTTCAGAGGAGAC	CTACAGCCTGGTATTTGG	XM_038715419.1
*zo1*	AATACACTCTCCCCAAAACGG	GCGAAGACCACGAAATCTCC	XM_038701018.1
*zo2*	GTCGTACCGCTCCTACTC	TTCTTGGTCCTCTATGCTC	XM_038733200.1
*caspase3*	GCTTCATTCGTCTGTGTTC	CGAAAAAGTGATGTGAGGTA	XM_038713063.1
*caspase9*	CTGGAATGCCTTCAGGAGACGGG	GGGAGGGGCAAGACAACAGGGTG	XM_038723308.1
*bad*	CACATTTCGGATGCCACTAT	TTCTGCTCTTCTGCGATTGA	Xie et al. (37)
*bax*	ACTTTGGATTACCTGCGGGA	TGCCAGAAATCAGGAGCAGA	Zhao et al. (39)
*bcl2*	CATCCTCCTTGGCTCTGG	GGGTCTGTTTGCCTTTGG	XM_038695757.1
*bag*	ATGACCCGAGACACGACAC	CATAACCTGGGCGAAGAAT	XM_038728315.1
*acaca*	ATCCCTCTTTGCCACTGTTG	GAGGTGATGTTGCTCGCATA	XM_038709737.1
*fasn*	TGTGGTGCTGAACTCTCTGG	CATGCCTAGTGGGGAGTTGT	XM_038735140.1
*scd*	CTGTGGGTGGCGTACTTCAT	TTGTCGTAGGGCCTGTTTCC	XM_038735580.1
*ppar*γ	CCTGTGAGGGCTGTAAGGGTTT	TTGTTGCGGGACTTCTTGTGA	XM_038695875.1
*srebp1*	AGTCTGAGCTACAGCGACAAGG	TCATCACCAACAGGAGGTCACA	XM_038699585.1
*atf6*	CAGGACGAAGTGCTTAGAGTT	AGAGTAATGGACGGTCACAAT	XM_038716053.1
*chopa*	GATGAGCAGCCTAAGCCACG	AACAGGTCAGCCAAGAAGTCG	XM_038701049.1
*chopb*	GTATCTTCATTACCAGTCCACCAG	AGGCGTTTCTTTGCTTTCC	XM_038721996.1
*eif2*α	CCTCGTTTGTCCGTCTGTATC	GCTGACTCTGTCGGCCTTG	XM_038693620.1
*perk*	ATTCTGGTTGATGAGCGGGC	GAAGGAGTTGGGGGTGTCTG	XM_038728395.1
*xbp1*	ACACCCTCGACACGAAAGA	AGAATGCCCAGTAGCAAATC	XM_038703562.1
*il1*β	CGTGACTGACAGCAAAAAGAGG	GATGCCCAGAGCCACAGTTC	Yu et al. (42)
*il8*	CGTTGAACAGACTGGGAGAGATG	AGTGGGATGGCTTCATTATCTTGT	Yu et al. (42)
*il10*	CGGCACAGAAATCCCAGAGC	CAGCAGGCTCACAAAATAAACATCT	Yu. et al. (42)
*il11*β	TTCCCAACAGACAGATGAAGAACTC	TGCCTGTGTTCAGCCAGTCAA	Yu et al. (42)
*tgf*β	GCTCAAAGAGAGCGAGGATG	TCCTCTACCATTCGCAATCC	Yu et al. (42)
*tnf*α	CTTCGTCTACAGCCAGGCATCG	TTTGGCACACCGACCTCACC	Yu et al. (42)
*cpt1a*	CATGGAAAGCCAGCCTTTAG	GAGCACCAGACACGCTAACA	Yu et al. (38)
*cpt2*	TGACCGTCACCTGTTTGCCAT	ATTGCAGCCGATCCAGTCGT	XM_038716807.1
*hsl*	AGGACAGGACAGTGAAGAGTTGC	CAGATAATTCTCATGGGATTTGG	Chen et al. (41)
*lpl*	ACCAGCACTACCCGACCTCC	CAGACTGTAACCCAGCAGATGAAT	XM_038715978.1
*gck*	CAGCGTGAGATGGACAGAGG	GGGGGTGGAGCAGACATAAG	XM_038703173.1
*ira*	CCCTTGTATCCCTCTCGTTT	CCAATTTCCTGTTCCTCTCC	XM_038717604.1
*pfkl*	CTGGCTGAGCTCGTAAAG	GTGCCGCAGAAGTCGTTG	Li et al. (40)
*pk*	CTCTTTCATCCGCAAAGC	AATTCCCAGGTCACCACG	Li et al. (40)
*gapdh*	CTGGTCATCGCTGGACAGAA	GCCTTCTCGATGGTGGTGAA	XM_038711150.1

*acaca*, acetyl-CoA carboxylase alpha; *atf6*, activating transcription factor 6; *bcl2*, B-cell lymphoma 2; *bad*, BCL2 associated agonist of cell death; *bag*, BCL2 associated athanogene 1; *bax*, BCL2 associated X; *chopa*, CCAAT enhancer binding protein homologous Protein a; *chopb*, CCAAT enhancer binding protein homologous Protein b; *eif2*α, GCN1 activator of EIF2AK4; *cpt1a*, carnitine palmitoyltransferase 1A; *cpt2*, carnitine palmitoyltransferase 2; *fasn*, fatty acid synthase; *gapdh*, glyceraldehyde-3-phosphate dehydrogenase; *gck*, glucokinase; *hsl*, hormone-sensitive lipase; *il1*β, interleukin 1 beta; *il8*, interleukin 8; *il10*, interleukin 10; *il11*β, interleukin 11 beta; *ira*, insulin receptor a; *lpl*, lipoprotein lipase; *perk*, eukaryotic translation initiation factor 2-alpha kinase 3; *pfkl*, phosphofructokinase, liver type; *pk*, pyruvate kinase; *ppar*γ, peroxisome proliferator activated receptor gamma; *scd*, stearoyl-CoA desaturase; *srebp1*, sterol regulatory element binding transcription factor 1; *tgf*β, transforming growth factor beta; *tnf*α, tumor necrosis factor alpha; *xbp1*, X-box binding protein 1; *zo1*, tight junction protein 1; *zo2*, tight junction protein 2.

### Plasma and tissue biochemical analyses

Plasma glucose level was measured by a commercial kit of LabAssayTM Glucose (Fujifilm, Wako Pure Chemical Corporation, Osaka, Japan). Plasma triglycerides (TG) levels were measured by a commercial kit of LabAssayTM Triglycerides (Fujifilm, Wako Pure Chemical Corporation, Osaka, Japan). Intestinal and hepatic triglycerides (TG) contents were measured by the Triglyceride Colorimetric Assay Kit (Cayman Chemical, Ann Arbor, MI, USA). The protein concentrations in the intestine and liver were detected by Bradford method (Beyotime, P0006, Shanghai, China) to normalize the content of tissue TG. All of the measurements were performed following the manufacturer’s instructions.

### Intestinal Caspase 3, Caspase 9, and plasma diamine oxidase determination

The activities of intestinal Caspase 3 and Caspase 9 were detected by the apoptosis detection kits (Beyotime, C1116 and C1158, Shanghai, China), according to the manufacturer’s instructions. The protein concentrations in the intestine were detected by Bradford method (Beyotime, P0006, Shanghai, China) to normalize the activities of Caspase 3 and Caspase 9. The activity of plasma diamine oxidase (DAO) was detected by the Micro Diamine Oxidase (DAO) Assay Kit (Solarbio, BC1285, Beijing, China), according to the manufacturer’s instruction.

### Oil red O staining, H&E staining, periodic acid-Schiff staining, and transferase dUTP nick end labeling staining

The intestine and liver sections initially fixed in 4% paraformaldehyde with 20% sucrose and sectioned with a cryostat (Thermo Fisher Scientific, Waltham, MA, USA), then the cryosections were stained with oil red O to visualize the lipid accumulation. After a 24-h fixation in 4% paraformaldehyde, the other intestine tissues were dehydrated in gradient alcohol dehydration, impregnated with xylene, embedded in paraffin and cut into 5 μm by Leica RM 2135 slicing machine (Leica Company, Wetzlar, Germany). Then, the intestine sections were stained with H&E or periodic Acid-Schiff (PAS) to visualize the tissue damages in the intestine. The intestine sections also stained with terminal deoxynucleotidyl transferase dUTP nick end labeling (TUNEL) to visualize the cell apoptosis in the intestine. Images were collected by Leica automatic digital slide scanner (Aperio VERSA 8, GER) and quantified with ImageJ (National Institutes of Health, USA).

### Transmission electron microscopy analysis

The transmission electron microscopy (TEM; HT-7700; Hitachi) was conducted according to the published methods (43, 44). In brief, after fixation (2.5% glutaraldehyde), samples were post-fixed in osmium tetroxide. The ultrathin sections were dehydrated in ethanol, embedded in resin, stained with uranyl acetate followed by leadcitrate, and then prepared for electron microscopy imaging.

### Immunofluorescence and imaging

Immunofluorescence on the intestine sections was performed as previously described (45). Primary antibodies were anti-IL-6 (Huabio, R1412-2, Rabbit), anti-BIP (Cell Signaling Technology, 3177, Rabbit), anti-SREBP1 (Abcam, ab28481, Rabbit), and anti-P-AMPKα (Cell Signaling Technology, 2535, Rabbit). Followed by Alexa Fluor 568 (Thermo Fisher Scientific, A11036, Rabbit) conjugated secondary antibody for visualization. Nuclei were stained with Hoechst 33342 (Thermo Fisher Scientific, H21492). Cell outlines were stained with phalloidin-488-iFluor (Yeasen Biotechnology, 40736ES75) for locating the intestinal villus. Images were collected using a Leica laser-scanning confocal microscope (SP8 DLS, GER), analyzed by Imaris Viewer (Oxford Instruments, UK), and quantified by ImageJ (National Institutes of Health, USA).

### Statistical analysis

All results are expressed as means ± SEMs (standard error of the mean). Normality was tested by 1-sample Kolmogorov–Smirnov test. Homogeneity of variance was examined by the Levene test. One-way ANOVA with Duncan’s multiple-range test was used to evaluate the significant differences in measured parameters among three groups. Differences with *P*-values < 0.05 were considered significant. All statistical analyses were carried out using GraphPad Prism 8 (GraphPad Software, San Diego, CA, USA).

## Results

### Dietary berberine improved the intestinal damages in largemouth bass fed with high carbohydrate diet

**FIGURE 1:**
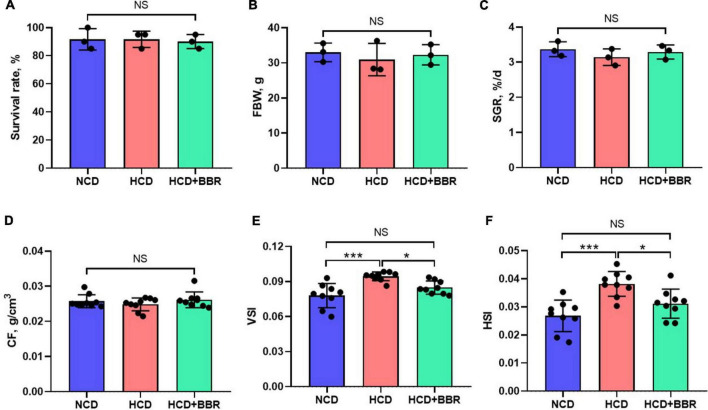
Effects of dietary BBR on the survival rate **(A)**, growth performance **(B,C)**, and physical indexes **(D–F)** of largemouth bass. Data are represented as means ± SEM [*n* = 3 for panels **(A–C)**, *n* = 9 for panels **(D–F)**]. *, ***Different from each other: *P < 0.05, ***P < 0.001, NS means no significant difference (One-way ANOVA, Duncan’s *post hoc* test).

**FIGURE 2:**
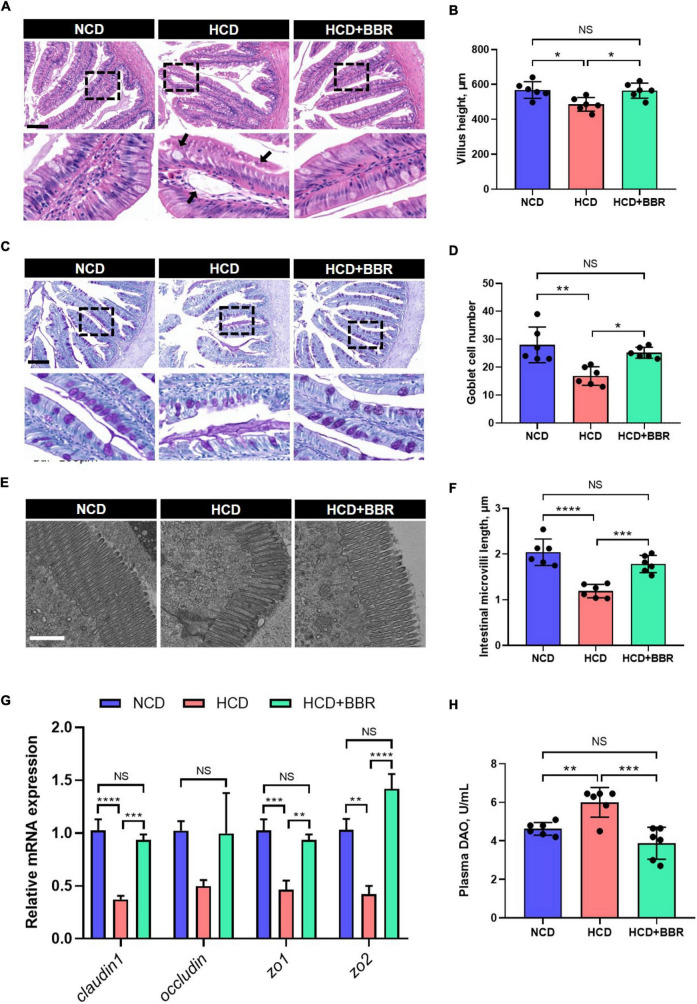
Dietary BBR improved the HCD-induced morphology and barrier damages in the intestine of largemouth bass. **(A)** H&E staining images of intestinal sections (arrows indicate damages, bar = 100 μm). **(B)** Villus height of intestine. **(C)** PAS staining images of intestinal sections (bar = 100 μm). **(D)** Goblet cell number in villus. **(E)** Transmission electron microscope images of intestinal microvilli (bar = 1 μm). **(F)** Intestinal microvilli length. **(G)** Expression level of tight junction-related genes. **(H)** Plasma DAO level. Data are represented as means ± SEM (*n* = 6). *, **, ***, ****Different from each other: *P < 0.05, **P < 0.01, ***P < 0.001, ****P < 0.0001, NS means no significant difference (One-way ANOVA, Duncan’s *post hoc* test).

After a 7-week feeding trial, there was no difference in survival rate, FBW, SGR and CF among the experimental groups (*P* > 0.05) (Figures 1A–D). However, HCD increased the VSI and HSI of largemouth bass (*P* < 0.05), while dietary BBR significantly decreased them to the similar levels of NCD (*P* < 0.05) (Figures 1E,F). These data suggest the innocuousness effects of 50 mg/kg dietary BBR in largemouth bass. Thus, we further investigated the pathological changes of intestine. Compared to NCD group, the villus height and goblet cell number were all significantly decreased in HCD group (*P* < 0.05), while they were restored by dietary BBR (Figures 2A–D). Besides, the H&E staining indicated that dietary BBR repaired the intestinal injury (Figure 2A). The transmission electron microscopy also showed that the intestinal microvilli length of HCD group was significantly lower than that of NCD group (*P* < 0.05), while it was comparable to NCD group under the treatment of dietary BBR (*P* > 0.05) (Figures 2E,F). Moreover, we evaluated the integrity of intestinal barrier by detecting the gene expression of occludin proteins. Compared to NCD group, we found that the expression levels of *claudin1*, *zo1*, and *zo2* were all significantly decreased in HCD group (*P* < 0.05), while they were all restored under the treatment of dietary BBR (Figure 2G). We further observed that the plasma DAO level was significantly increased in HCD group (*P* < 0.05) but reduced to the level of NCD group with the dietary BBR (*P* > 0.05) (Figure 2H). These results suggest that dietary BBR improves the HCD-induced intestinal damages of largemouth bass.

### Dietary berberine suppressed high carbohydrate diet-induced apoptosis *vi*a alleviating inflammation and endoplasmic reticulum stress in the intestine of largemouth bass

**FIGURE 3:**
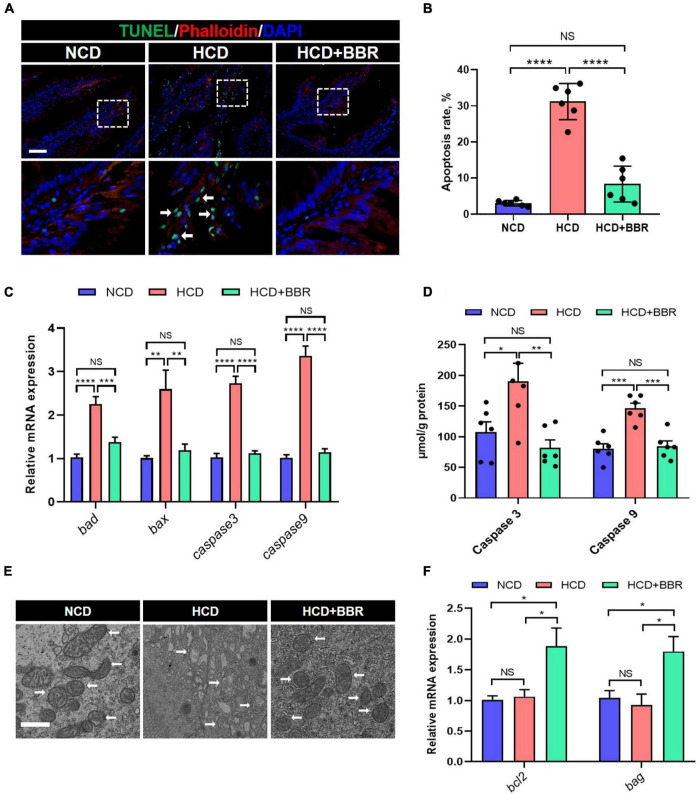
Dietary BBR suppressed the HCD-induced apoptosis in the intestine of largemouth bass. **(A)** TUNEL (green) staining images of intestinal sections (arrows indicate TUNEL signal, bar = 50 μm). **(B)** Apoptosis rate in intestinal sections. **(C)** Expression level of proapoptotic genes. **(D)** Intestinal Caspase 3 and Caspase 9 level. **(E)** Transmission electron microscope images of intestinal mitochondria (arrows indicate mitochondria, bar = 1 μm). **(F)** Expression level of anti-apoptotic genes. Data are represented as means ± SEM (*n* = 6). *, **, ***, ****Different from each other: *P < 0.05, **P < 0.01, ***P < 0.001, ****P < 0.0001, NS means no significant difference (One-way ANOVA, Duncan’s *post hoc* test).

Cell apoptosis is one of the main causes for tissue damages (46). Given dietary BBR improved the intestinal injury with the HCD stimulation, we investigated the apoptosis status in the intestine of largemouth bass. The results showed that HCD markedly increased the intestinal apoptosis rate (*P* < 0.05), while dietary BBR significantly suppressed the HCD-induced intestinal apoptosis (*P* < 0.05) (Figures 3A,B). Accordingly, the elevated expression level of the intestinal proapoptotic genes (*bad*, *bax*, *caspase3*, and *caspase9*) in HCD group was all significantly decreased under the treatment of dietary BBR (*P* < 0.05) (Figure 3C). The activity levels of intestinal Caspase3 and Caspase9 showed similar changes with the proapoptotic genes (*P* < 0.05) (Figure 3D). We also observed mitochondrial damage in the intestinal transmission electron microscopy of HCD group (Figure 3E). Besides, the expression level of anti-apoptotic genes (*bcl2* and *bag*) was exclusively elevated in the dietary BBR group (*P* < 0.05) (Figure 3F). These data indicate that dietary BBR suppresses HCD-induced intestinal apoptosis.

**FIGURE 4:**
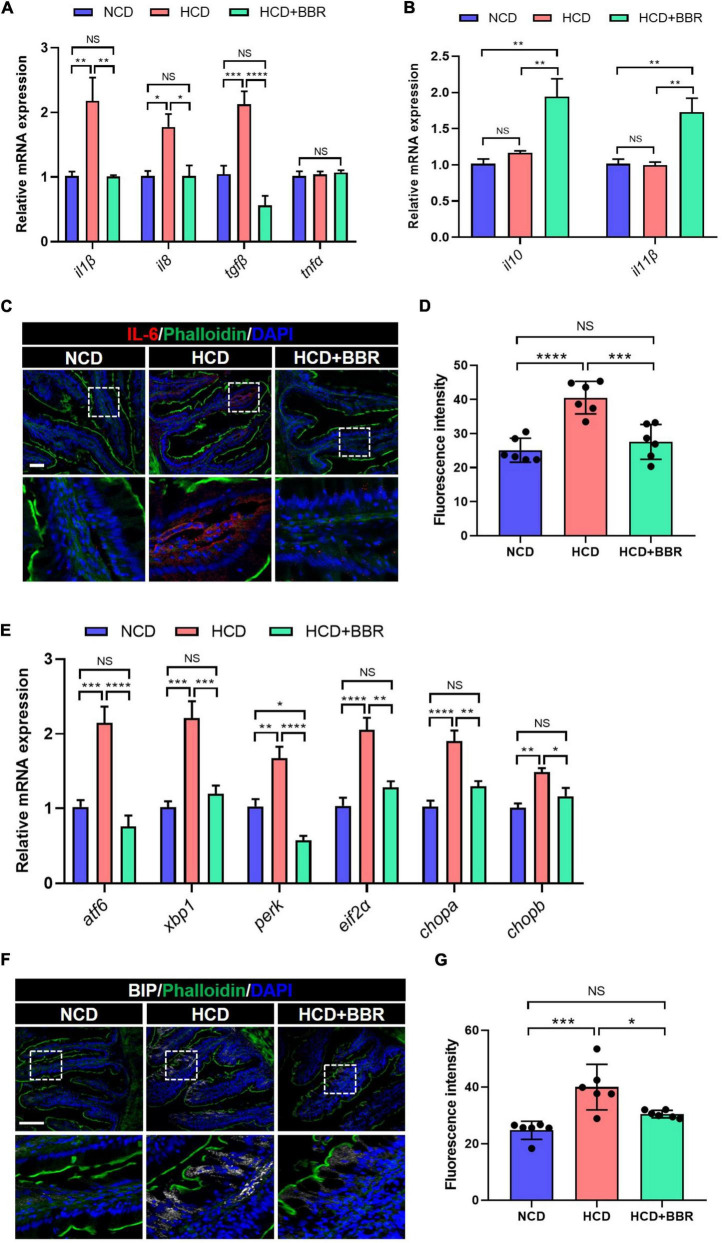
Dietary BBR alleviated the HCD-induced inflammation and ER stress in the intestine of largemouth bass. **(A)** Expression level of proinflammatory genes. **(B)** Expression level of anti-inflammatory genes. **(C)** IL-6 (red) staining images of intestinal sections (bar = 50 μm). **(D)** Quantification of the IL-6 fluorescence intensity. **(E)** Expression level of ER stress-related genes. **(F)** BIP (white) staining images of intestinal sections (bar = 100 μm). **(G)** Quantification of the BIP fluorescence intensity. Data are represented as means ± SEM (*n* = 6). *, **, ***, ****Different from each other: *P < 0.05, **P < 0.01, ***P < 0.001, ****P < 0.0001, NS means no significant difference (One-way ANOVA, Duncan’s *post hoc* test).

Since apoptosis is usually induced by consistent inflammation and ER stress (47, 48), we further evaluated the inflammation and ER stress status in the intestine of largemouth bass. The results showed that the expression level of proinflammatory genes (*il1*β, *il8*, and *tgf*β) was significantly elevated in HCD group (*P* < 0.05), but suppressed by dietary BBR (*P* < 0.05) (Figure 4A). However, the expression level of anti-inflammatory genes (*il10* and *il11*β) was only elevated in the dietary BBR group (*P* < 0.05) (Figure 4B). Accordingly, we also observed that the protein level of IL-6 was increased in HCD group (*P* < 0.05), while it was decreased to a comparable level to NCD group by dietary BBR (Figures 4C,D). Moreover, the expression level of ER stress-related genes (*atf6*, *xbp1*, *perk*, *eif2*α, *chopa*, and *chopb*) was also significantly elevated in HCD group (*P* < 0.05), while it was almost decreased to the similar level of NCD by dietary BBR (*P* > 0.05) (Figure 4E). The protein level of BIP, an ER stress maker, was significantly increased by HCD but suppressed by dietary BBR (*P* < 0.05) (Figures 4F,G). These data suggest that dietary BBR alleviates HCD-induced intestinal apoptosis *via* inhibiting inflammation and ER stress in largemouth bass.

### Dietary berberine reduced the intestinal lipid deposition *via* regulating AMPK/SREBP1 cascade in largemouth bass fed with high carbohydrate diet

**FIGURE 5:**
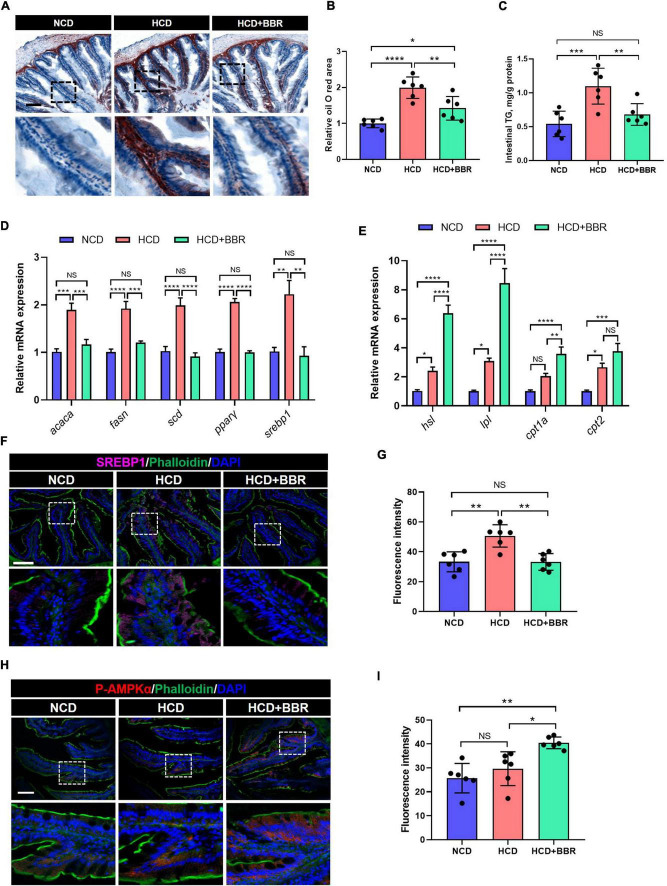
Dietary BBR reduced the HCD-induced excessive lipogenesis and promoted lipid catabolism *via* activating AMPK and inhibiting SREBP1 in the intestine of largemouth bass. **(A)** Oil Red O staining images of intestinal sections (bar = 100 μm). **(B)** Relative oil Red O area in intestinal sections. **(C)** Intestinal TG level. **(D)** Expression level of lipogenesis genes. **(E)** Expression level of lipolysis and fatty acid oxidation genes. **(F)** SREBP1 (pink) staining images of intestinal sections (bar = 100 μm). **(G)** Quantification of the SREBP1 fluorescence intensity. **(H)** P-AMPKα (red) staining images of intestinal sections (bar = 100 μm). **(I)** Quantification of the P-AMPKα fluorescence intensity. Data are represented as means ± SEM (*n* = 6). *, **, ***, ****Different from each other: *P < 0.05, **P < 0.01, ***P < 0.001, ****P < 0.0001, NS means no significant difference (One-way ANOVA, Duncan’s *post hoc* test).

Long-term HCD induced chronic inflammation and ER stress *via* over-deposition of lipid (8, 11). Using Oil Red O staining, we observed that the intestinal lipid was significantly increased in the HCD group (*P* < 0.05), while dietary BBR significantly decreased the HCD-induced over-deposition of lipid (*P* < 0.05) (Figures 5A,B). The intestinal TG level exhibited a similar change among the three groups (Figure 5C). We further found that the expression level of lipogenesis genes (*acaca*, *fasn*, *scd*, *ppar*γ, and *srebp1*) was also significantly elevated by HCD (*P* < 0.05), but significantly decreased by dietary BBR (*P* < 0.05) (Figure 5D). Moreover, the expression level of lipolysis and fatty acid oxidation genes (*hsl*, *lpl*, *cpt1a*, and *cpt2*) was markedly elevated by dietary BBR (*P* < 0.05), though some of them (*hsl*, *lpl*, and *cpt2*) also showed a mild but significant increase in HCD group (*P* < 0.05) (Figure 5E). These data indicate that dietary BBR reduce the intestinal lipid deposition *via* suppressing lipogenesis and activating lipid catabolism. Interestingly, the protein level of SREBP1 was also significantly increased in the HCD group (*P* < 0.05), while decreased with the presence of dietary BBR (*P* < 0.05) (Figures 5F,G). Importantly, we further observed that the protein level of p-AMPKα was exclusively increased by dietary BBR (*P* < 0.05) (Figures 5H,I). These results suggest that dietary BBR improves the intestinal lipid metabolism disorder *via* activating AMPK and inhibiting SREBP1 in the largemouth bass.

### Dietary berberine promoted hepatic lipid catabolism and glycolysis in largemouth bass fed with high carbohydrate diet

**FIGURE 6:**
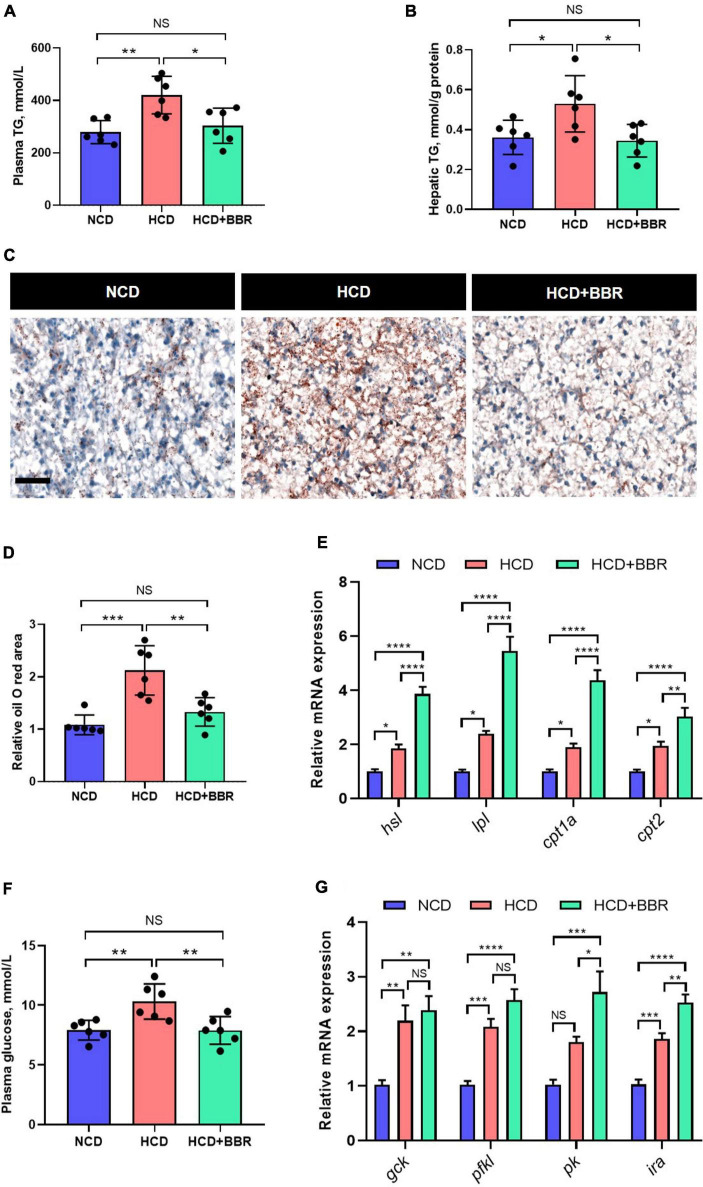
Dietary BBR promoted the lipid catabolism and glycolysis in the liver of largemouth bass fed with HCD. **(A)** Plasma TG level. **(B)** Hepatic TG level. **(C)** Oil Red O staining images of liver sections (bar = 50 μm). **(D)** Relative oil Red O area in liver sections. **(E)** Expression level of hepatic lipolysis and fatty acid oxidation genes. **(F)** Plasma glucose level. **(G)** Expression level of hepatic glycolysis genes. Data are represented as means ± SEM (*n* = 6). *, **, ***, ****Different from each other: *P < 0.05, **P < 0.01, ***P < 0.001, ****P < 0.0001, NS means no significant difference (One-way ANOVA, Duncan’s *post hoc* test).

Aside from the intestine, liver is the central organ for systemic lipid and glucose metabolism (49). Therefore, we further investigated the effects of dietary BBR on the hepatic lipid and glucose metabolism. We found that the HCD-induced excessive TG levels of plasma and liver were both significantly normalized by dietary BBR (*P* < 0.05) (Figures 6A,B). The Oil Red O staining of liver showed that HCD significantly increased the hepatic lipid deposition (*P* < 0.05), while dietary BBR decreased that to the level of NCD group (Figures 6C,D). The expression level of lipolysis and fatty acid oxidation genes (*hsl*, *lpl*, *cpt1a*, and *cpt2*) showed a mild but significant increase in HCD group (*P* < 0.05), while it was further elevated by dietary BBR (*P* < 0.05) (Figure 6E). Besides, the plasma glucose level was also significantly normalized by dietary BBR from the HCD-induced hyperglycemia (*P* < 0.05) (Figure 6F). Accordingly, we found that the expression level of glycolysis genes (*gck*, *pfkl*, and *ira*) was significantly increased in HCD group and dietary BBR group (*P* < 0.05), while the expression levels of *pk* and *ira* were further elevated by dietary BBR compared to HCD group (*P* < 0.05) (Figure 6G). These data suggest that dietary BBR promotes the hepatic lipid catabolism and glycolysis of largemouth bass fed with HCD.

**FIGURE 7:**
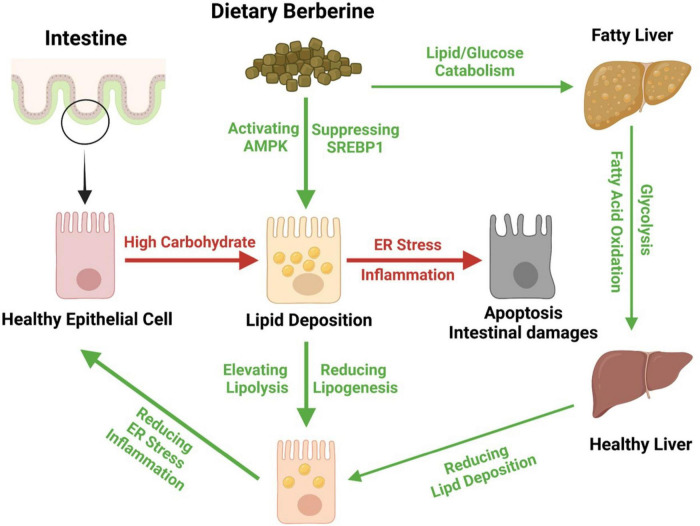
Proposed working model depicting dietary berberine alleviates HCD-induced intestinal damages *via* improving lipid metabolism in largemouth bass.

Together, we found that dietary BBR (50 mg/kg) improved the HCD-induced apoptotic damages and repaired the barrier by alleviating the inflammation and ER stress in the intestine of largemouth bass. Besides, we further found that dietary BBR suppressed the HCD-induced intestinal lipid deposition *via* regulating the AMPK/SREBP1 cascade, and it also promoted the hepatic lipid catabolism and glycolysis to reduce the systematic lipid deposition in largemouth bass (Figure 7).

## Discussion

Carbohydrates are a large category of aquafeeds ingredients, which is of lower price and necessary for puffing (1–3). However, long-term HCD intake insults the intestinal health of aquaculture species *via* series of metabolism disorders (9–11). Berberine is isolated from Chinese herbs (21), which has been shown to improve glucose and lipid metabolism, enhance insulin sensitivity and defend oxidative stress (25–27). Therefore, here we investigated the effects of dietary BBR on improving the HCD-induced intestinal damages in largemouth bass. We demonstrated that dietary BBR (50 mg/kg) alleviated HCD-induced intestinal damages *via* improving the intestinal and hepatic lipid metabolism in largemouth bass.

Excessive ingestion of dietary carbohydrate not only leads to hepatic steatosis (8, 50), but also significantly impairs the intestinal metabolism and physiology in fish (9–11). BBR is a potential candidate for alleviating the HCD-induced metabolism disorders and tissue damages (24–26, 30). Previous studies showed that dietary carbohydrate decreased the intestinal villus height in yellow catfish (11) and largemouth bass (51). In the present study, we did observe that HCD shorten the intestinal villus and microvilli; however, they were all restored by dietary BBR. These results indicated that dietary BBR protected intestinal villus form HCD-induced atrophy in fish. Interestingly, we also observed that dietary BBR suppressed the decline of goblet cell under the HCD treatment. Although a study in largemouth bass also showed HCD decreased the number of goblet cell (51), we investigated the effect of dietary BBR on preserving fish goblet cell in the present study. Moreover, we found that the dietary BBR preserved the integrity of intestinal barrier (transcription level of *claudin1*, *occluding*, *zo1* and *zo2*, and plasma DAO level). Previous study indicated that chronic HCD activated apoptosis, that caused tissue pathological deterioration whether in fish or mammalian (9, 52, 53). Therefore, we speculated that these intestinal damages attributed to the hyperactivation of apoptosis. Consistently, we observed HCD induced a marked intestinal apoptosis, while dietary BBR reduced it by suppressing the proapoptotic process and elevating the anti-apoptotic process in the present study. A few studies demonstrated that BBR suppressed the apoptosis in cardiomyocyte (54) and podocyte (55). Thus, we supposed that this anti-apoptotic effect of BBR was reserved in the intestine of largemouth bass fed with HCD.

Cell apoptosis is tightly associated to chronic inflammation and ER stress (47, 48). Thus, we detected the inflammation and ER stress status to further decipher the underlying mechanism for BBR alleviating intestinal damages. In aquaculture species, HCD induced hepatic inflammation and apoptosis in blunt snout bream (52), and stimulated the transcription of intestinal proinflammatory genes in largemouth bass (51). In the present study, we also found that HCD induced a severe intestinal inflammation, which was characterized by elevated the proinflammatory factors (*il1*β, *il8*, *tgf*β, and IL6). Despite the similar observations in other fish (10, 51), our results are also consistent with a study in rodent (56). Importantly, we found that dietary BBR significantly improved the HCD-induced inflammation by suppressing the proinflammatory factors and stimulating the anti-inflammatory factors (*il10* and *il11*β) in the intestine of largemouth bass. These data are partially supported by the studies of BBR in both mammalian and fish (27, 29). Moreover, the HCD-induced intestinal ER stress, characterized by elevated ER stress makers (*atf6*, *xbp1*, *perk*, *eif2*α, *chopa*, *chopb*, and BIP), was significantly alleviated by dietary BBR in largemouth bass. It has been demonstrated that BBR improves tissue injury *via* alleviating ER stress in the liver and brain of mice (57, 58); however, there is few study investigates the effects of dietary BBR on improving HCD-induced intestinal ER stress before. Besides, a study in hepatocytes indicated that BBR inhibited free fatty acid and LPS-induced inflammation through modulating ER stress response (59). Although a previous study demonstrated that BBR alleviate intestinal damages injured by high-fat or high-carbohydrate diet *via* modulating intestinal microflora in fish (22), here we proposed that dietary BBR decreased the intestinal apoptosis mainly through the anti-inflammation and anti-ER stress effects in the largemouth bass fed with HCD.

Chronic HCD triggers supraphysiological lipogenesis and cellular lipid deposition (8, 60), that results in lipotoxic inflammation and ER stress (61, 62). In the present study, we found that dietary BBR decreased the HCD-induced lipid accumulation *via* suppressing the lipogenesis and activating the lipolysis in the intestine of largemouth bass. AMPK pathway is well-known to regulate the lipid metabolism (63). Interestingly, here we found that dietary BBR significantly elevated the phosphorylation level of AMPK (P-AMPKα). AMPK pathway is the one of the major targets of BBR (64), that also negatively regulates SREBP1 (38, 63). Given the decreased SREBP1 signal in this study, we supposed that the BBR-activated AMPK signal inhibited the SREBP1-mediated lipid anabolism and promoted the lipid catabolism in the intestine of largemouth bass. Furthermore, we found that the dietary BBR also reduced the HCD-induced hepatic lipid accumulation by activating lipid catabolism in largemouth bass. Since the liver is the central organ for lipid metabolism (65), we proposed that the increased hepatic lipid catabolism relieved the peripheric lipid accumulation. Besides, previous study indicated that BBR could improve the blood glucose hemostasis *via* promoting insulin secretion (24). Consistently, we observed that the dietary BBR normalized the hyperglycemia by partially stimulating the glycolysis (transcription level of *pk* and *ira*) in the largemouth bass fed with HCD. These results suggested that the dietary BBR improved hepatic lipid and glucose metabolism, which contributed to deceasing the intestinal lipid deposition. Besides, previous study also indicated that dietary BBR inhibited hepatic glycogen synthesis to reduce the hepatic metabolic burden in blunt snout bream (66). This is also of interest for further investigations.

## Conclusion

In the present study, we investigated the effects and mechanism of dietary BBR on alleviating the HCD-induced intestinal damages in largemouth bass. We found that dietary BBR (50 mg/kg) reduced the intestinal morphology damages and repaired the intestinal barrier by suppressing the inflammation and ER stress in the largemouth bass fed with HCD. We further interpreted that dietary BBR reduced excessive lipogenesis and promoted the lipid catabolism *via* regulating AMPK/SREBP1 cascade to cope with the HCD-induced intestinal inflammation and ER stress in largemouth bass. Besides, we also found that dietary BBR promoted the hepatic fatty acid oxidation and glycolysis to reduce the systematic lipid deposition in largemouth bass fed with HCD. These findings propose that BBR is a feasible aquafeed additive for preserving the intestinal health in aquaculture species.

## Data availability statement

The original contributions presented in this study are included in the article/supplementary material, further inquiries can be directed to the corresponding author.

## Ethics statement

All animal experiments were performed according to the Guide for Animal Care and Use Committee of Institute of Hydrobiology, Chinese Academy of Sciences (IHB, CAS, Protocol No. 2016–018).

## Author contributions

DH: conceptualization. YG, QL, LX, and YL: investigation. HL and YY: methodology. YG and DH: writing. JJ, ZZ, XZ, and SX: writing, review, and editing. All authors contributed to the article and approved the final version.
